# Roost selection by Mauritian tomb bats (*Taphozus mauritianus*) in Lilongwe city, Malawi – importance of woodland for sustainable urban planning

**DOI:** 10.1371/journal.pone.0240434

**Published:** 2020-11-05

**Authors:** Kieran D. O’Malley, William E. Kunin, Matthew Town, William O. Mgoola, Emma Louise Stone

**Affiliations:** 1 School of Biological Sciences, University of Leeds, Leeds, United Kingdom; 2 African Bat Conservation, Lilongwe, Malawi; 3 Department of National Parks and Wildlife Malawi, Lilongwe, Malawi; 4 Bat Conservation Research Lab, Department of Applied Sciences, University of the West of England, Bristol, United Kingdom; University of Western Ontario, CANADA

## Abstract

Increasing urbanisation has led to a greater use of artificial structures by bats as alternative roost sites. Despite the widespread presence of bats, roost availability may restrict their distribution and abundance in urban environments. There is limited quantitative information on the drivers of bat roost selection and roosting preferences, particularly in African bats. We explore the factors influencing roost selection in the Mauritian tomb bat (*Taphozous mauritianus*), within an urban landscape in Lilongwe city, Malawi. Eight building and five landscape features of roosts were compared with both adjacent and random control buildings throughout the city. Bat occupied buildings were situated closer to woodland (mean 709m) compared to random buildings (mean 1847m) but did not differ in any other landscape features explored. Roosts were situated on buildings with larger areas and taller walls, suggesting bats select features for predator-avoidance and acoustic perception when leaving the roost. Bats preferred buildings with exposed roof beams which may provide refuge from disturbance. Whilst roosts are situated more often on brick walls, this feature was also associated with landscape features, therefore its importance in roost selection is less clear. These results are indicative that *T*. *mauritianus* selects roosts at both the building and landscape level. The selectivity of *T*. *mauritianus* in relation to its roost sites implies that preferred roosts are a limited resource, and as such, conservation actions should focus on protecting roost sites and the woodland bats rely on.

## Introduction

Urban areas are expanding at an unprecedented rate, causing significant reductions in biodiversity and ecosystem service provision [1]. Sixty-eight percent of the world's population is projected to live in cities by 2050, an increase of 13% from 2018 estimates [2]. Anthropogenic activities are a major threat to bats globally and more than a third of bat species are considered threatened or data deficient by the International Union for Conservation of Nature (IUCN) [3]. Factors threatening bat populations include habitat loss and fragmentation, roost disturbance, agriculture, hunting, persecution, non-native predators, urban development, climate change, and the emergence of diseases such as white-nose syndrome in North America [3–6].

Bats spend over half their time under the influence of the selective pressures imposed upon them by their roost environment [7]. Roosts provide bats with important sites for hibernation, mating, and rearing young; as well as offering protection from environmental extremes and predators [8]. For many bats, roosts often play a significant part in shaping and maintaining social interactions [9, 10] and the type and location of available roosts is likely to play a decisive role in determining bat survival and fitness [11].

The conversion of natural landscapes into agricultural land and managed forests has meant many bats are forced to use human-made structures due to a lack of natural roost sites [12]. Some bats have benefited from the increased roosting opportunities provided by human development [13–17]. However, the lower intensity of urban habitat use across bat species compared to natural areas indicates that overall bats are affected negatively by urbanisation [18]. Increased human-bat conflict (HBC), opportunistic predators, a reduction in health through pollutants, and artificial lighting have all been suggested as potential barriers to colonisation of urban areas by bats [19, 20]. The ability of bats to respond to urbanisation is highly dependent on the mobility, degree of specialisation, mobility, behavioural plasticity and the spatial scale at which species respond [21, 22]. Thus, a species may react positively, negatively, or in a neutral way to urban encroachment depending on the circumstances.

To date, relatively few studies have investigated the process of roost selection in bats occupying buildings [15, 23–26]. Distance to woodland and water, coverage at roost sites, linear vegetation elements, and temperature have all been shown to influence roost selection for several bat species [15, 27]. This reliance on both local and landscape-scale elements means the size and distribution of bat populations may be constrained by the availability of favorable roost sites. Bat species that utilise a narrow range of resources for roosting and foraging may find it difficult to adapt to future urban expansion.

Globally, most urbanisation occurs in developing countries, which are predicted to contain 83% of the world’s urban population by 2050 [2]. More than half of global population growth (58%) by 2050 is expected to occur in Africa [2]. Although the vast majority of bat species diversity resides within the tropics [28], to date no studies have investigated the drivers of roost selection of tropical bats in an urban environment. This lack of information limits our ability to develop effective mitigation strategies for HBC and sustainable management of biodiversity in increasingly urbanised environments. Understanding roost selection and the degree of ecological specialisation by bats can assist in determination of species vulnerability to habitat loss and climate change, informing global assessments of species conservation status [29, 30].

We assessed the drivers of roost selection in the Mauritian tomb bat *Taphozous mauritianus* in Lilongwe city, Malawi. *T*. *mauritianus* is a small and widespread species that roosts under the eaves of buildings in central and south-eastern Africa [31]. Whilst being listed as a species of least concern, the IUCN identifies a need to elucidate this bat’s population size, distribution, and dynamics. This species often roosts in buildings in Malawi which increases HBC. An improved understanding of its roosting habits is critical to inform conflict mitigation and determine species distribution over large geographic areas [32].

To date only basic descriptive studies on the roost sites of *T*. *mauritianus* are available [33–35] limiting our understanding of the roosting requirements of this species and therefore effective management of roosts, habitats and HBC. We used a paired occupied and unoccupied control design to assess predictors of roost occupancy at the local and landscape scale. We hypothesised that *T*. *mauritianus* select roost sites based on both the local-scale features of buildings and the surrounding landscape matrix. At the landscape scale, we predicted that *T*. *mauritianus* select roosts situated close to available resources, such as woodland and water bodies, in accordance with previous studies on other species [15, 24, 27]. At the local scale, we predicted *T*. *mauritianus* select roosts situated away from any direct sunlight [33], closer to trees, and with larger building eaves than unoccupied sites.

## Materials and methods

### Study species

The Mauritian tomb bat is a species of sac-winged bat in the family Emballonuridae (body mass ~ 26g) which occurs throughout central and south-eastern Africa and Madagascar [36]. *T*. *mauritianus* are most commonly found in moist, open habitats and savanna regions [37], but also in open woodland [33]. *T*. *mauritianus* primarily roost with their abdomen against a vertical surface. Day roosts are commonly found in the open but away from direct sunlight, and include the outer walls of buildings, cliff faces, and the trunks of large trees [33]. *T*. *mauritianus* can tolerate high levels of human disturbance but remain constantly vigilant during the day. *T*. *mauritianus* are listed as least concern due to their wide distribution, presumed large population, and tolerance to a degree of habitat modification [37].

### Study area

We conducted the study within the residential zones of Lilongwe city, in central Malawi (13°58’60”S, 33°46’60”E, 1,050 m above sea level) during the dry season months of June to July 2018. Malawi is located in Southern Africa with an estimated population of 17.5 million which is expected to double by 2018 [38]. Lilongwe is the Capital city of Malawi, covering an area of 456 km2 with an estimated population of 989,318, and population density of 2455/km^2^ in 2018 [39]. Urbanisation is increasing in Lilongwe, with a 50% increase in area of settlements between 2008 and 2017 [40]. Anthropogenic habitats constitute 79.64% of land area (e.g. subsistence agriculture and unwooded urban areas), with only 20.36% of the city containing natural habitats (woodland, grassland, shrub land, parkland, open water and dambo). The majority of land use in Lilongwe is low intensity agriculture (52.3%) followed by high density unwooded urban area (15.27%), medium density wooded urban areas (8.87%) and unwooded urban areas (8.09%) (S1 Table) [41]. Roosts were located within an area of approximately 29 km^2^ situated to the north of Lilongwe River characterized by high and medium urban density areas, with low levels of woodland.

### Roosts and random buildings

We identified twenty-one bat-occupied buildings (BOBs) through door-to-door surveys within the study area. We systematically inspected all walls at each roost and visually counted bats. We chose thirteen random unoccupied buildings as control samples by selecting the nearest house to a randomly generated grid reference acquired using QGIS 2.2.0 [42]. If building access was not possible, we assessed the next closest building until access was granted. We externally inspected buildings to confirm the absence of bats. Due to the external roosting habits of *T*. *mauritianus*, confirmation of the absence of bats could be assessed with absolute certainty. All buildings used in the study were occupied by humans. We were only able to obtain access to thirteen unoccupied buildings due to logistical constraints.

### Paired controls

Roost selection of bats found in optimal habitat could be due to specific building features, or the process could be more akin to random selection [15, 23, 43]. To assess relative importance of building features we identified 21 paired control buildings by selecting the nearest unoccupied building to the BOB (defined as < 200 m away [15, 43]). If access to the nearest unoccupied building was not possible, the owners of the next closest building were contacted until access was granted.

### Building features

We recorded the following features for all sampled buildings (e.g. bat-occupied, random and paired controls): (i) height of wall ((m) at bat roost locality), (ii) wall material (brick or non-brick), (iii) roof material (corrugated metal or tiles), (iv) eaves depth (defined as the length from the end of the eaves to the point where the eaves met the wall (m)), (v) wall orientation (north, north-east, east, south-east, south, south-west, west, north-west), (vi) building area (m^2^), (vii) building perimeter (m), (viii) presence of exposed roof beams and (ix) distance from roost to the nearest tree greater than 5 m tall. Measurements were carried out using a Bosch GLM 250 VF Professional laser range finder and a Garmin eTrex® 10 handheld GPS unit.

We allocated a number to the wall of each control building and used a random number generator to select a wall from which the building measurements were recorded. For paired buildings, the number of walls examined equaled the number measured on the paired BOB. For random controls, we took measurements on up to a maximum of six walls. We took all measurements on control buildings from the central point of each wall selected. We conducted research under permit from the Department of National Parks and Wildlife Malawi.

### Landscape features

We quantified habitats around roost and random control buildings in QGIS 2.2.0 [42] using aerial photographs obtained from Google Satellite imagery (dated: 29/05/2018). We created habitat land cover maps using 17 pre-defined habitat/land use categories (Table 1) which we digitised from Google satellite imagery using the Open Layers plugin within QGIS version 2.16.3. We digitised habitat polygons visually from spatial data at scales between 1:7,000 and 1:10,000 and which were ground validated by researchers at African Bat Conservation (ABC). We recorded the following landscape features: (i) distance to nearest woodland (or parkland) (m), (ii) distance to nearest open water (river, lake, swimming pool, reservoir, pond, and fountain) (m), (iii) area of nearest woodland (or parkland) (ha), (iv) road density (sum length [m] of road within 500 m), (v) and building density. We measured building density by counting the number of buildings within 1.5km of each building in the following concentric distance bands: 0–0.5 km, 0.5–1.0 km, and 1.0–1.5 km using Google Satellite Imagery.

**Table 1:** Top models (Δ_i_ < 2) predicting building occupancy by *T*. *mauritianus* based on AIC_C_ and Akaike weight W_i_ for comparisons between: (i) roosts and all controls (paired + random) and (ii) roosts and paired controls.

Model	AIC_C_	D_i_	W_i_
***Roosts vs all controls***			
**Wall height + area + wall material**^***a***^ **+ eaves depth**^***a***^ **+ beams**^***a***^	49.2728	0.0000	0.2176
**Wall height + area + wall material**	49.6630	0.3901	0.1790
**Wall height + area + wall material + eaves depth**^***a***^ **+ beams**^***a***^ **+ tree**^***a***^	50.4491	1.1762	0.1209
**Wall height + area + eaves depth + beams**	50.8716	1.5987	0.0978
**Intercept only**	174.8033	125.5305	0.0000
***Roosts vs paired***			
**Wall height + area + beams**	32.5679	0.0000	0.5639
**Wall height + area + beams + eaves depth**^***a***^	34.3921	1.8242	0.2265
**Intercept only**	122.6432	90.0752	0.0000

Models built with binomial distribution and logit link. Intercept models included for comparisons under conditions of no roost selection. D_i_ represents delta (AIC_C_) and indicates the difference in the AIC_C_ value with the top model. The symbol *‘a’* denotes non-significant terms based on 0.05 significance threshold.

### Statistical analysis

We used an information theoretic approach to assess the relative importance of characteristics for roost selection by *T*. *mauritianus* [44]. We developed candidate models using building and landscape characteristics. We analysed explanatory variables for collinearity using pearsons correlations, and assessed multicollinearity by calculating the variance inflation factor (VIF) for each variable within a given model (a value exceeding four indicated significant multicollinearity [45]). To eliminate variable redundancy, we dropped building perimeter from analyses as it was highly correlated with building area (kendall’s tau = 0.8, p < 0.0001).

We used Generalized Linear Models (GLMs) with a binomial error structure and a logit link function to assess the role of building and landscape features on occupancy of buildings by *T*. *mauritianus*. We assigned a value of 1 to BOBs and 0 to random/paired bat-unoccupied buildings (response variable). Building and landscape features were incorporated as fixed effects (explanatory variables). We developed separate models to assess the importance of factors important for local (i.e., roost height, eaves depth, wall and roof material, orientation, exposed beams, distance to tree, building area) and landscape features (i.e., distance to woodland and water, area of nearest woodland, road and building density).

To assess the importance of building features in roost selection, we pooled datasets for random and paired buildings (i.e., all controls) and compared with BOBs. We compared features of BOBs with paired bat-unoccupied buildings to determine the local features important for roost selection, independent of landscape features. For the assessment of building features, the roost, or wall from which the measurements were taken from on the paired buildings, was the unit under investigation within the developed models. We compared BOBs to random bat-unoccupied buildings to investigate whether bats selected buildings according to local landscape features.

Model fit was analysed using Akaike Information Criterion scores corrected for small sample sizes (AIC_C_), as well as Akaike weights (W_i_). The difference in AIC_C_ between the *i*th and top-ranked model (Δ_i_) was evaluated. Models in which Δ_i_ < 2 received substantial support and were so considered the top selected models [43]. Where multiple models received substantial support (i.e., Δ_i_ < 2), we summed Akaike weights for each model in which a particular variable occurred (abbreviated as W_+_) to aid interpretation [42–44]. This method allows assessment of the relative importance of any given variable and is recommended when many models are investigated [44]. All analyses were conducted within the RStudio environment [46] and graphical outputs created with OriginPro [47].

## Results

We recorded a total of 75 individual *T*. *mauritianus* on 21 separate buildings including churches, universities, and residential buildings in Lilongwe (S1 Table). All *T*. *mauritianus* roosts were situated on the outside of buildings, exclusively under the eaves. Bats consistently roosted on buildings in which the roof beams were exposed and avoided the apex side of buildings.

### Building features

#### Roost vs all controls

We pooled data from random and paired buildings (i.e., all controls), and no single best model could be inferred (Table 1). There was no difference in eaves depth or presence of beams between BOBs and control buildings (lowest AIC_C_ model; eaves: X^2^ = 1.232, df = 1, p = 0.267; beams: X^2^ = 3.267, df = 1, p = 0.071).

In all top models, bat-occupied buildings were larger and walls were taller compared to unoccupied ones (lowest AIC_C_ model; height: X^2^ = 14.910, df = 1, p < 0.001; area: X^2^ = 9.630, df = 1, p = 0.002) (Table 1). Occupied buildings were 216.59 m^2^ larger (mean ± SE, roosts: 486.89 *±* 33.63 m^2^; controls: 229.72 ± 13.24 m^2^), and walls 2.17 m taller (mean ± SE, roosts: 5.61 ± 0.21 m; controls: 3.50 *±* 0.04 m), compared to control buildings.

Wall material was not a predictor of building occupancy (X^2^ = 3.785, df = 1, p = 0.053) (Table 1), though this was only marginally insignificant. Assessment of the relative importance of parameters indicates that wall material is an important feature predicting bat presence (Table 2). Bats selected buildings constructed of brick (90.9% of roost walls constructed with brick compared to 42.5% for controls). The intercept only model scored one of the highest AIC_C_ values (ranked 100 out of 104), indicating that bats are highly selective of roosts based on building features.

**Table 2:** The relative importance (W_+_) of variables in predicting the presence of *T*. *mauritianus* on buildings throughout Lilongwe, Malawi.

Scale	Variable	W_+_
**Building (*roosts vs all controls*)**	Building area	0.9534
	Wall height	0.9201
	Wall material	0.8076
	Eaves depth	0.5846
	Beams	0.4888
	Distance to tree	0.2730
	Roof material	0.0769
	Orientation	0.0000
**Building (*roosts vs paired*)**	Building area	0.9923
	Beams	0.9728
	Wall height	0.9594
	Eaves depth	0.3792
	Wall material	0.1614
	Distance to tree	0.0476
	Roof material	0.0066
	Orientation	0.0000
**Landscape (*roosts vs random*)**	Distance to woodland	1.2037
	Building density (0–0.5 km)	0.8055
	Road density	0.4173
	Distance to water	0.4061
	Building density (1.0–1.5 km)	0.3854
	Woodland area	0.2924
	Building density (0.5–1.0 km)	0.2232

‘W_+_’ represents the sum of Akaike weights for each model the variable appears in. Models built with binomial distribution and logit link.

#### Roosts vs paired controls

Of 106 models comparing BOBs with paired buildings two models were supported (Δ_i_ < 2, W_**+**_ = 0.7904) (Table 2). Both top candidate models were consistent with previous models (i.e., roosts vs all controls) as roost height (X^2^ = 9.994, df = 1, p = 0.002) and building area (X^2^ = 24.822, df = 1, p < 0.001) predicted occupancy by *T*. *mauritianus*. The presence of beams also determined bat occupancy (X^2^ = 12.287, df = 1, p < 0.001).

Wall material was not found to be a predictor of building occupancy by bats and had low relative importance when controlling for landscape features (Table 2). The intercept only model scored one of the highest AIC_C_ values, indicating that building features are playing an important role in the roost selection process of *T*. *mauritianus* when in an optimal habitat.

### Landscape features

#### Roosts vs random controls

To assess the impact of landscape features on building occupation by *T*. *mauritianus*, we compared 141 models using AIC_C_ and Akaike weight values (Table 3). No single best model could be determined from the analyses because the top ten models had a Δ_i_ < 2 and a cumulative Akaike weight of 0.424 (Table 3). All top candidate models agreed that BOBs were situated closer to woodland compared to non-occupied random controls (lowest AIC_C_ model; X^2^ = 14.408, df = 1, p < 0.001). BOBs were an average of 1131.73 m closer to woodland than unoccupied buildings (mean ± SE, roosts: 708.59 ± 178.08 m; controls: 1840.32 ± 162.06 m). No other landscape variables affected roost selection of *T*. *mauritianus*. Evaluation of the relative importance of each landscape feature indicated strong support that distance to woodland had a greater importance over any other landscape variable (Table 2). The intercept-only model was not included in the confidence set of models, receiving little support (Δ_i_ = 12.1457 and W_i_ = 0.0002), and can thus be discounted as a plausible model.

**Table 3:** Top models (Δ_i_ < 2) assessing the impact of landscape features on building occupancy by *T*. *mauritianus* based on AIC_C_ and Akaike weight W_i_. Results based on comparisons made between roosts and random controls.

Model	AIC_C_	D_i_	W_i_
**Distance to woodland**	35.2132	0.0000	0.0693
**Distance to woodland + building density (0.5 km)**^***a***^ **+ building density (1500m)**^***a***^	35.9262	0.7130	0.0485
**Distance to woodland + distance to water**^***a***^ **+ road density (0.5 km)**^***a***^ **+ building density (0.5 km)**^***a***^	36.1329	0.9197	0.0437
**Distance to woodland + woodland area**^***a***^	36.1679	0.9546	0.0430
**Distance to woodland + road density**^***a***^	36.4442	1.2310	0.0374
**Distance to woodland + distance to water**^***a***^ **+ density of buildings (0.5 km)**^***a***^	36.4653	1.2520	0.0370
**Distance to woodland + density of buildings (0.5 km)**^***a***^	36.7091	1.4959	0.0328
**Distance to woodland + distance to water**^***a***^ **+ road density**^***a***^ **+ building density (500m)**^***a***^ **+ building density (1.0–1.5 km)**^***a***^	36.8790	1.6657	0.0301
**Distance to woodland + distance to water**^***a***^ **+ building density (500m)**^***a***^ **+ building density (1.0–1.5 km)**^***a***^	37.0187	1.8055	0.0281
**Distance to woodland + building density (1.0–1.5 km)**^***a***^	37.0822	1.8690	0.0272
**Distance to woodland + distance to water**^***a***^	37.0955	1.8822	0.0270
**Intercept only**	47.3589	12.1457	0.0002

Models built with binomial distribution and logit link. Intercept model included for comparisons under conditions of no roost selection. D_i_ represents delta (AIC_C_) and indicates the difference in the AIC_C_ value with the top model. The symbol *‘a’* denotes non-significant terms based on 0.05 significance threshold.

## Discussion

Here we show that *T*. *mauritianus* are highly selective of roosts based on building features and local landscape variables. At the building level, we predicted *T*. *mauritianus* would select roosts situated away from any direct sunlight [33], and with larger building eaves than unoccupied buildings. Indeed in our study, *T*. *mauritianus* preferred buildings with taller walls and larger areas. This is consistent with studies characterising roost preferences in other bat species, despite the differences in their roosting ecology [14, 23–25, 48]. Bats in rural Madagascar selected larger and taller buildings as roosts [14]. Tree-roosting bats tend to choose taller trees—an apparent mechanism of predator-avoidance from terrestrial predators such as weasels [11, 49]. Bats tend to favour structures that are significantly taller than surrounding structures [23–25, 48]. A study in South Africa recorded *T*. *mauritianus* roosting at an average height of around six meters, though it was not clear whether bats were actively selecting taller buildings [35].

Roosting at height may reduce the risk of predation, either by reducing the risk of discovery from ground predators or by increasing the difficulty of them climbing up [11]. Whilst predators within urban areas may vary from those in non-urban areas, the challenges presented may be somewhat similar. Domestic cats are often the most prevalent predator of bats within cities, particularly for bats roosting in houses [50]. Cats are common in Lilongwe city along with other natural predators including genets (*Genetta genetta*) and snakes. As such, *T*. *mauritianus* may be under a selective pressure to roost at height to avoid predation. Roosting externally on buildings makes *T*. *mauritianus* easily visible to humans and therefore roosting at height may also reduce human disturbance and HBC. Studies of attic dwelling bats suggested that a preference for tall buildings reduces the risk of exclusion [24]. Roosting at height may also allow for ease of take-off, and improved orientation as taller dominant objects provide better acoustic and visual perception cues when returning from foraging [48].

In accordance with our predictions *T*. *mauritianus* preferred larger buildings compared to controls. House dwelling bats in Madagascar also preferred larger buildings [14]. A larger building area may provide more opportunities for roost sites, especially as larger buildings often become increasingly complex in architectural design.

We predicted that *T*. *mauritianus* would select buildings located close to trees, as previous observations suggested that *T*. *mauritianus* sometimes flies to nearby trees when disturbed or threatened [33, 51]. This was not supported by our results, as *T*. *mauritianus* roosts were not situated closer to trees compare to control buildings. During anecdotal observations we found that disturbed bats would seek refuge in other areas on the building rather than a nearby tree. This, combined with the preference for the presence of exposed beams, suggests *T*. *mauritianus* may use exposed beams for refuge when disturbed rather than flying to nearby trees. This contrasts with previous findings for tree-roosting bats in natural day roosts, which showed a preference for roost sites situated close to trees [11]. Therefore, buildings may provide greater refuge opportunities than those naturally available, and is likely to increase with building size.

Although wall material was not a significant predictor of occupancy for paired buildings, wall material was in the top three highest ranked models when roosts were compared to all controls. Therefore, whilst bats may prefer brick structures (as found by bats in Madagascar [14]), it is possible that bats select buildings due to location and or wider landscape features, and that brick buildings are more commonly found in the areas with preferred landscape. Nevertheless, 91% of occupied roosts were comprised of brick. This is consistent to findings from an observational study of *T*. *mauritianus* in Durban, South Africa where around 80% of roosts were associated with brick walls [34, 35]. Whilst brick structures may provide an easier surface for bats to grip, they may provide camouflage due to the grizzled pelage of *T*. *mauritianus* [52]. Consequently, bats may select brick surfaces as they resemble natural surfaces such as cliff walls and tree trunks [33], compared to the majority of non-brick buildings which had white painted walls, which would make *T*. *mauritianus* conspicuous when roosting. The spatial distribution of bats and occupation of roosts is not solely dependent on building or landscape features, but also influenced by the degree of roost fidelity and social cohesion. In Lilongwe there is evidence that *T*. *mauritianus* exhibits high roost fidelity (at least one roost has been occupied by bats for 10 years). High roost fidelity and preference for certain structural features indicates that some buildings would be suboptimal roost sites, and thus roost sites may be a limiting resource over time due to changes in urban buildings making *T*. *mauritianus* vulnerable to disturbance and roost loss.

In line with previous studies, we predicted that bats would select roost sites based on their proximity to resources such as water and foraging sites [15, 24, 25, 27, 43, 53]. Indeed some bats select roosts closer to open water than random buildings [15, 27]. In this study, roost buildings were not situated closer to open water compared to unoccupied buildings, indicating bats are not selecting roosts according to proximity to riparian habitats. However, *T*. *mauritianus* have been recorded foraging over water [54, 55], suggesting the lack of preference for roost sites close to water could reflect the homogeneity of open water features throughout Lilongwe and the similarity in proximity to water between roost and random buildings sampled (mean distance to water roost buildings = 415 m, unoccupied buildings = 494 m).

*T*. *mauritianus* selected roosts situated close to woodland. Previous observations of *T*. *mauritianus* within open and riparian forest suggest a level of dependency on these habitat types [33]. Fragmented patches of woodland provide an important habitat for moths in urban environments by allowing them to proliferate [56]. Therefore, it is possible *T*. *mauritianus* occupying roosts in urban areas may rely on woodland habitat for foraging [33]. This highlights the importance of maintaining urban woodlands for bats, as despite being considered “urban exploiters” the ability of *T*. *mauritianus* to occupy urban areas is dependent upon not only the presence of preferred buildings, but also access to woodlands for foraging. Malawi has the highest rate of deforestation in the Southern African Development Community (SADC) region (estimated at 30,000–40,000 hectares per year), due to agricultural expansion, development and fuel use [57]. Woodland in Lilongwe declined by 25% between 1990 and 2010, and is under increasing threat from continued residential and industrial development, and fuel wood extraction [41]. Deforestation in Lilongwe and other tropical cities threatens the persistence of “urban exploiter” bats, and may render urban areas unsuitable for bats. Conservation of remaining urban woodland should therefore be prioritised in urban biodiversity management plans.

## Conclusions and conservation implications

Urbanisation is a major threat to biodiversity globally, yet many species may benefit from it, including bats [58]. Our results demonstrate patterns of non-random association between *T*. *mauritianus* and building and landscape features which is indicative of roost selection. We have provided the first quantified evidence of the specific building and roost structural features preferred by *T*. *mauritianus*, information which is critical to inform roost mitigation, creation and conservation in urban areas. The preference for larger buildings with taller walls, containing exposed beams will be important information in managing HBC and roost conservation in tropical areas. Perhaps most significantly, we have demonstrated the importance of nearby woodland for urban roost occupancy by *T*. *mauritianus*, which could make this species vulnerable to future urbanisation. Lilongwe city is rapidly expanding and woodlands are declining, which may reduce the suitability of urban areas for *T*. *mauritianus* whilst simultaneously increasing HBC. *T*. *mauritianus* are not legally protected in Malawi and are subject to persecution, resulting in the destruction of entire colonies (ABC pers com.).

Effective conservation of *T*. *mauritianus* in urban areas will therefore require the protection of roosts, conservation of urban woodlands and public education about the ecological importance of bats within the urban landscape [59]. Our results will inform practical measures to mitigate HBC with *T*. *mauritianus* and inform effective conservation of bats and their habitats in urban environments across their range.

## Supporting information

S1 Dataset Building features. (CSV)

S2 Dataset Landscape features. (CSV)

S1 Table Description of habitat categories in Lilongwe, Malawi. (DOCX)

S2 Table Description of occupied and unoccupied buildings in Lilongwe, Malawi. (DOCX)
