# Supplementary material for: Roost selection by Mauritian tomb bats (Taphozus mauritianus) in Lilongwe city, Malawi – importance of woodland for sustainable urban planning
Source: PLoS One. 2020 Nov 5;15(11):e0240434. doi: 10.1371/journal.pone.0240434 (PMC7644015; doi:10.1371/journal.pone.0240434)
Supplement: S1 Table — (DOCX) [file pone.0240434.s003.docx]

| S1 Table. Description of habitat categories in Lilongwe, Malawi. | | |  |
| --- | --- | --- | --- |
| **Category** | **Description** | **Area (ha)** | **%Coverage** |
| **Low Density Urban - No wooded** | Mixture of constructed materials and vegetation; Constructed materials = 10%-40%, Vegetation = 50%-70% of the cover, with no/few trees/shrubs | 1407 | 3.22 |
| **Low Density Urban - Wooded** | Mixture of constructed materials and vegetation; Constructed materials = 10%-40%, Vegetation = 50%-70% of the cover, with trees/shrubs present | 751 | 1.72 |
| **Medium Density Urban - No wooded** | Mixture of constructed materials and vegetation; Constructed materials = 30%-80%, Vegetation = 20%-60% of the cover, with no/few trees/shrubs | 3536 | 8.09 |
| **Medium Density Urban - Wooded** | Mixture of constructed materials and vegetation; Constructed materials = 30%-80%, Vegetation = 20%-60% of the cover, with trees/shrubs present | 3880 | 8.87 |
| **High Density Urban - No wooded** | Highly developed areas where people reside in high numbers; Constructed materials = 70%-100%, Vegetation < 20% of the cover, with no/few trees/shrubs | 6680 | 15.27 |
| **High Density Urban - Wooded** | Highly developed areas where people reside in high numbers; Constructed materials = 70%-100%, Vegetation < 20% of the cover, with trees/shrubs present | 1290 | 2.95 |
| **Low Intensity Agriculture** | Areas of scattered crops and fields, small holder plantations | 22,891 | 52.34 |
| **Medium-High Intensity Agriculture** | Intense row cropping, industrial farms | 317 | 0.72 |
| **Parkland** | Manicured open park areas containing low density mature trees | 91 | 0.21 |
| **Monoculture non-native woodland** | Plantations of non-native species | 16 | 0.04 |
| **Mixed Nonnative woodland** | Non-native woodland including Maligna, bamboo, etc. | 111 | 0.25 |
| **Mixed Miombo Woodland** | Native woodland, with few non natives | 73 | 0.17 |
| **Shrubland** | Low shrubs, mixed with grasses and herbaceous vegetation | 1854 | 4.24 |
| **Grassland/Scrub** | Grasses, flowers and herbs | 750 | 1.71 |
| **Riparian Woodland** | Hydrophilic plant communities adjacent to water | 66 | 0.15 |
| **Dambo** | Seasonally flooded/wet grassland | 3 | 0.01 |
| **Open water** | Open Water | 18 | 0.04 |
