# Supplementary material for: Roost selection by Mauritian tomb bats (Taphozus mauritianus) in Lilongwe city, Malawi – importance of woodland for sustainable urban planning
Source: PLoS One. 2020 Nov 5;15(11):e0240434. doi: 10.1371/journal.pone.0240434 (PMC7644015; doi:10.1371/journal.pone.0240434)
Supplement: S2 Table — (DOCX) [file pone.0240434.s004.docx]

**S2 Table. Description of occupied and unoccupied buildings in Lilongwe, Malawi.**

| **Variable** | **Roosts** | **Paired controls** | **Random controls** |
| --- | --- | --- | --- |
| **Building features** | | | |
| **Wall height** | Median = 5.92 m  Mean = 5.62 m  Range = 3.15 – 9.54 m | Median = 3.58 m  Mean = 3.56 m  Range = 2.98 – 4.18 m | Median = 3.43 m  Mean = 3.45 m  Range = 2.32 – 4.57 m |
| **Wall material** | 90.90% brick  9.10% non-brick | 74.42% brick  25.58% non-brick | 15.69% brick  84.31% non-brick |
| **Roof material** | 36.36% corrugated metal  63.64% tiles | 95.35% corrugated metal  4.65% tiles | 100% corrugated metal |
| **Presence of beams** | 100% yes | 76.74% yes  23.26% no | 80.39% yes  19.61% no |
| **Eaves depth** | Median = 1.05 m  Mean = 1.12 m  Range = 0.28 – 3.44 m | Median = 0.65 m  Mean = 0.69 m  Range = 0.18 – 1.42 m | Median = 0.64 m  Mean = 0.67 m  Range = 0.21 – 1.34 m |
| **Building area** | Median = 440.27 m^2^  Mean = 486.89 m^2^  Range = 162.87 – 1124.91 m^2^ | Median = 162.4 m^2^  Mean = 181.58 m^2^  Range = 97.17 – 695.30 m^2^ | Median = 245.42 m^2^  Mean = 270.30 m^2^  Range = 113.63 – 517.05 m^2^ |
| **Distance to tree** | Median = 11.44 m  Mean = 13.38 m  Range = 4.42 – 35.28 m | Median = 9.75 m  Mean = 10.75 m  Range = 2.92 – 29.19 m | Median = 11.90 m  Mean = 11.39 m  Range = 2.08 – 21.23 m |
| **Orientation** | 11.36% north  15.91% north-east  9.09% east  15.91% south-east  4.55% south  6.82% south-west  13.64% west  20.45% north-west | 6.98% north  13.95% north-east  2.33% east  25.58% south-east  4.65% south  16.28% south-west  6.98% west  23.26% north-west | 15.69% north  11.76% north-east  11.76% east  5.88% south-east  15.69% south  11.76% south-west  13.73% west  13.73% north-west |
| **Landscape features** | | | |
| **Distance to open water** | Median = 157.54 m  Mean = 190.18 m  Range = 16.73 – 842.11 m | Median = 152.54 m  Mean = 245.67 m  Range = 28.36 – 964.27 m | Median = 162.71 m  Mean = 217.21 m  Range = 12.26 – 525.03 m |
| **Distance to woodland** | Median = 195.12 m  Mean = 708.59 m  Range = 8.96 – 2561.27 m | Median = 89.90 m  Mean = 699.53 m  Range = 34.07 – 2519.50 m | Median = 1854.28 m  Mean = 1840.32 m  Range = 1146.99 – 3061.65 m |
| **Area of woodland** | Median = 1.75 km^2^  Mean = 1.28 km^2^  Range = 0.17 – 1.75 km^2^ | Median = 1.24 km^2^  Mean = 1.17 km^2^  Range = 0.09 – 1.75 km^2^ | Median = 1.75 km^2^  Mean = 1.17 km^2^  Range = 0.09 – 1.75 km^2^ |
| **Road density** | Median = 4698.45 m  Mean = 4904.79 m  Range = 3049.41 – 6371.05 m | Median = 4639.03 m  Mean = 4839.29 m  Range = 3457.12 – 6568.87 m | Median = 6248.14 m  Mean = 5987.53 m  Range = 2828.97 – 7293.17 m |
| **Building density (0.5 km)** | Median = 305  Mean = 303.80  Range = 218 – 475 | Median = 328  Mean = 317.68  Range = 178 – 470 | Median = 306  Mean = 315.46  Range = 107 – 525 |
| **Building density (0.5-1.0 km)** | Median = 864.50  Mean = 883.81  Range = 393 – 2029 | Median = 780  Mean = 834.15  Range = 423 – 1998 | Median = 771  Mean = 860.46  Range = 467 – 1419 |
| **Building density (1.0-1.5 km)** | Median = 1063.50  Mean = 1183.67  Range = 570 – 3195 | Median = 1069  Mean = 1194.60  Range = 652 - 3028 | Median = 1140  Mean = 1140.08  Range = 801 - 3064 |
